# Genome Sequence of *Formosa haliotis* Strain MA1, a Brown Alga-Degrading Bacterium Isolated from the Gut of Abalone *Haliotis gigantea*

**DOI:** 10.1128/genomeA.01312-16

**Published:** 2016-11-17

**Authors:** Reiji Tanaka, Yukino Mizutani, Toshiyuki Shibata, Hideo Miyake, Shunpei Iehata, Tetsushi Mori, Kouichi Kuroda, Mitsuyoshi Ueda

**Affiliations:** a Laboratory of Marine Microbiology, Mie University, Kurima-machiya, Tsu, Mie, Japan; b School of Fisheries and Aquaculture Science, University Malaysia, Kuala Terengganu, Terengganu, Terengganu, Malaysia; c Graduate School of Advanced Science and Engineering, Waseda University, Shinjuku, Tokyo, Japan; d Division of Applied Life Science, Graduate School of Agriculture, Kyoto University, Kitashirakawa-oiwake, Sakyo, Kyoto, Japan; e Japan Science and Technology Agency, CREST, Chiyoda-ku, Tokyo, Japan

## Abstract

*Formosa haliotis* is a brown alga-degrading bacterium isolated from the gut of abalone *Haliotis gigantea*. Here, we report the draft genome sequence of this bacterium and pointed out possible important features related to alginate degradation.

## GENOME ANNOUNCEMENT

Alga-degrading bacteria are considered important targets recently since they harbor significant genes for the assimilation of alginate, a major component for the production of bioethanol from brown algae (1, 2). These bacteria are also important for evolutionary studies to understand the yet-unclear mechanisms behind alginate assimilation (3). Here, we report the draft genome sequence of a novel brown alga-degrading bacterium, *Formosa haliotis* strain MA1 (LMG 28520^T^), isolated from the gut of abalone *Haliotis gigantea* collected from Mie Prefecture, Japan. Previously, we classified this bacterium as a new species of the genus *Formosa* (4).

The genome of this bacterium was sequenced by shotgun sequencing using the Pac Bio RS2 system (Pacific Biosciences). Sequence runs were conducted on bacterial genome extracted on separate occasions, and 173,359 sequence reads were attained from both runs (average read length, 4,030 bp). Sequencing reads were pooled and *de novo* assembled using the SMRT Analysis version 2.3 (Pacific Biosciences) (5) to reveal a total of 4,305,801 bp, with an average G+C content of 34.36%, consisting of one contig. Automatic gene annotation was performed by the Rapid Annotations using Subsystems Technology (RAST) (6), and an overview of the annotated genome was viewed using the SEED Viewer (7). The genome sequence contains 3,760 coding sequences (CDSs), of which 2,076 CDSs (52%) were classified in 361 subsystems, while 1684 CDSs (48%) were uncategorized. In addition, 46 tRNA genes for 21 amino acids and 12 rRNA genes (short subunit [SSU], four; long subunit [LSU] four; 5S, four) were identified.

Analyzing the pathways within the draft genome, we determined the alginate degradation gene cluster. Two coding sequences (CDS nos. 1087 and 1099) were identified to encode alginate lyase classified to the CAZy (8) alginate lyase family PL-7. Three CDSs (1364, 3150, and 3156) were identified to encode oligoalginate lyase classified to the alginate lyase family PL-17. We found that oligoalginate lyase formed a small operon with several ABC transporter-related genes (3153 and 3154), DEH reductase (3159), KdgF (3152), and 2-keto-3-deoxy-d-gluconate (KDG) kinase (3166). The 2-dehydro-3-deoxy-phosphogluconate (KDPG) aldolase (688) gene was located in a separate area. Several candidate CDSs distributed throughout the genome showing similarity to genes found within the alginolytic clusters of *Formosa agariphila* were also identified (9). We believe that the genome information of *Formosa haliotis* strain MA1 may serve as an important resource for research in alginate degradation and saccharification processes.

### Accession number(s).

This whole-genome shotgun project has been deposited at DDBJ/EMBL/GenBank under the accession no. BDEL00000000. The version described in this paper is version BDEL01000000.
